# Reservoirs of Red-Spotted Grouper Nervous Necrosis Virus (RGNNV) in Squid and Shrimp Species of Northern Alboran Sea

**DOI:** 10.3390/v14020328

**Published:** 2022-02-06

**Authors:** Carolina Johnstone, Montse Pérez, Marta Arizcun, Cristina García-Ruiz, Elena Chaves-Pozo

**Affiliations:** 1 Oceanographic Center of Málaga, Spanish Institute of Oceanography, Spanish National Research Council, Puerto Pesquero s/n, Fuengirola, 29640 Málaga, Spain; carolina.johnstone@ieo.es (C.J.); cristina.garcia@ieo.es (C.G.-R.); 2 Oceanographic Center of Vigo, Spanish Institute of Oceanography, Spanish National Research Council, Subida a Radio Faro 50, Vigo, 36390 Pontevedra, Spain; montse-perez@ieo.es; 3 Oceanographic Center of Murcia, Spanish Institute of Oceanography, Spanish National Research Council, Carretera de la Azohía s/n, Puerto de Mazarrón, 30860 Murcia, Spain; marta.arizcun@ieo.es

**Keywords:** betanodavirus, shrimp, squid, Alboran Sea, host-driven distribution

## Abstract

The production of the aquaculture industry has increased to be equal to that of the world fisheries in recent years. However, aquaculture production faces threats such as infectious diseases. Betanodaviruses induce a neurological disease that affects fish species worldwide and is caused by nervous necrosis virus (NNV). NNV has a nude capsid protecting a bipartite RNA genome that consists of molecules RNA1 and RNA2. Four NNV strains distributed worldwide are discriminated according to sequence homology of the capsid protein encoded by RNA2. Since its first description over 30 years ago, the virus has expanded and reassortant strains have appeared. Preventive treatments prioritize the RGNNV (red-spotted grouper nervous necrosis virus) strain that has the highest optimum temperature for replication and the broadest range of susceptible species. There is strong concern about the spreading of NNV in the mariculture industry through contaminated diet. To surveil natural reservoirs of NNV in the western Mediterranean Sea, we collected invertebrate species in 2015 in the Alboran Sea. We report the detection of the RGNNV strain in two species of cephalopod mollusks (*Alloteuthis media* and *Abralia veranyi*), and in one decapod crustacean (*Plesionika heterocarpus*). According to RNA2 sequences obtained from invertebrate species and reported to date in the Mediterranean Sea, the strain RGNNV is predominant in this semienclosed sea. Neither an ecosystem- nor host-driven distribution of RGNNV were observed in the Mediterranean basin.

## 1. Introduction

The quest for sustainable fisheries and procurement of food supply has increased the production of the aquaculture industry to the same level as the world fisheries [1]. However, aquaculture production faces threats such as infectious diseases. Betanodaviruses induce a neurological disease (VER, viral encephalopathy and retinopathy) that affects fish species worldwide, the disease outbreaks being more frequent among early developmental stages (larval and juveniles) [2]. The disease caused by the betanodavirus Nervous necrosis virus (NNV) is known as viral encephalopathy and retinopathy (VER) or viral nervous necrosis (VNN), and was described over 30 years ago [3]. Since then, the virus has expanded (177 susceptible marine species) and reassortant strains have appeared [2,4].

The International Committee on Taxonomy of Viruses classifies NNV in the family *Nodaviridae*, which has two genera, *Alfabetanodavirus* that infect insects and *Betanodavirus* that infect fish species, and includes NNV [5]. Nowadays, betanodaviruses are classified into four recognized strains, although three additional strains have been suggested [2]. In addition, a new *Gammanodavirus* genera has been proposed to include crustacean betanodaviruses causing a certain degree of disease [6,7]. No clear evidence of disease is found for most of the invertebrate hosts in which betanodaviruses have been detected, thus these species are considered virus carriers or reservoirs. Up to date, betanodaviruses have been detected in 21 species of marine invertebrates belonging to 12 families and nine orders [2]. The NNV virion has a proteic nude capsid protecting a bipartite RNA viral genome that consists of positive-stranded molecules RNA1 and RNA2. Proteins related to viral replication, including RNA-dependent RNA polymerase (RdRp), are transcribed from RNA1, whereas RNA2 encodes the structural protein of the capsid, the C or coat protein (CP). The four recognized betanodavirus strains are discriminated according to sequence homology of RNA2 encoding the coat protein; SJNNV (Striped jack nervous necrosis virus), TPNVV (Tiger puffer nervous necrosis virus), BFNNV (Barfin flounder nervous necrosis virus)*,* and RGNNV (Redspotted grouper nervous necrosis virus) [8]. Reassortment of RNA1 and RNA2 gene fragments of different strains results in reassortant strains, and an example is the RGNNV/SJNNV reassortant, currently a threat for the marine aquaculture industry in the Mediterranean [9].

Regarding the transmission of NNV, viral particles have been reported to be stable for months in a wide range of experimentally simulated marine environmental conditions [10]. In fish species, the virus is transmitted horizontally from fish to fish or through the water body, and transmission occurs between different species [2]. Moreover, vertical transmission through the gonads has also been reported in fish [11]. Concerning invertebrate species, infectious particles have been recovered from seawater [12] and clam tissues [13]. Interestingly, clams are also able to release infective viral particles through feces and filtered water [13]. Reservoirs in invertebrate species may partly explain the somewhat latent behavior of NNV in certain ecosystems, and the appearance of recurrent outbreaks of VER disease. Furthermore, the infection of fish species through feeding with trash fish or contaminated diet has raised strong concern. Experimental infection of invertebrate cultures (Artemia and Rotifers) used as food in aquaculture has been reported [14], and recently, disease was recorded after feeding Senegalese sole larvae with NNV-infected Artemia [15]. Trash fish have been demonstrated to act as vehicles for NNV infection in cultured marine fish [16]. Thus, evidence points to the necessity of identifying invertebrate reservoirs of NNV, characterizing them and also determining their role in epidemiology and risk for marine ecosystem services.

Reservoirs of NNV have been reported in a considerable number of invertebrate marine species. The first studies were reported in South Korea in apparently healthy commercial aquarium invertebrates [17]. In coastal waters of the Korean peninsula, wild marine crab, shrimp and mussel species were found to host the RGNNV strain [18]. Positive samples with homology to RGNNV were also reported in Japanese coastal waters, specifically in squid captured to feed cultured marine fish [16]. Recent studies document the presence of RGNNV and BFNNV in marine bivalve mollusks collected in waters of South Korea, Japan and China [19]. In European Atlantic and Mediterranean waters, the detection of NNV reservoirs in wild marine invertebrate species has also been reported. Major presence of the RGNNV strain in bivalve mollusks was reported in the Atlantic coast of France [20]. Regarding the Mediterranean sea, RGNNV has been detected in bivalve mollusks in the coast of Italy [20], in the Adriatic Sea [21] and in Greece [22]. A recent study on bivalve mollusks in the Adriatic Sea reported apparent RGNNV/SJNNV reassortants, although RGNNV was the main strain detected [9]. As for other invertebrate species, NNV was also documented in Italy in naturally deceased specimens of cultured and wild octopus presenting skin lesions [23], in Greece in gastropod samples [22] and in specimens of the blue crab from the Levantine Sea [24]. The present study aimed to contribute to the surveillance of reservoirs of NNV in the unexplored western Mediterranean Sea, reporting non-natural reservoirs of the RGNNV strain in squid and shrimp species collected in the northern Alboran Sea (ALB).

## 2. Materials and Methods

### 2.1. Field Collection of Samples of Invertebrate Species in the ALB

**Figure 1:**
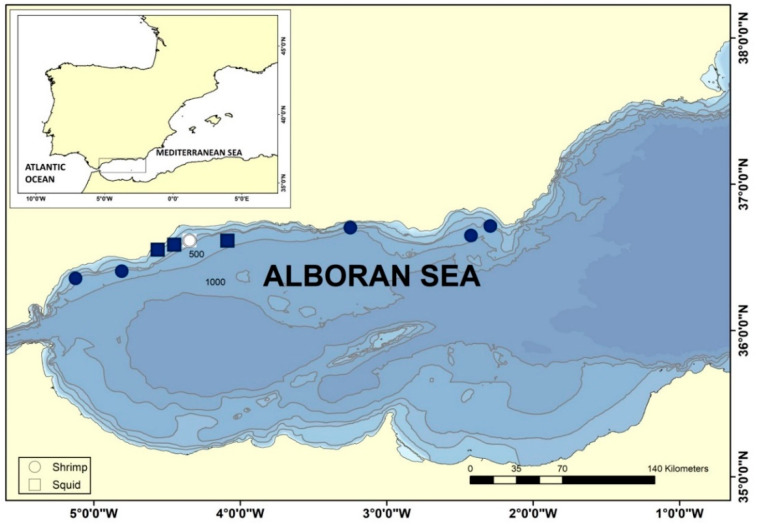
Collection of shrimp and squid species in the Alboran Sea during *MEDITS* survey, 2015. Specimens were collected in nine locations along the coast and frozen upon retrieval. Circles refer to trawls in which the shrimp *Plesionika heterocarpus* was captured and squares refer to trawls in which squid *Alloteuthis media* and *Abralia veranyi* were collected. Color-filled symbols indicate detection of RGNNV in RNA extracted from pooled ocular tissue of captured shellfish.

Specimens of squid and shrimp were collected in the ALB in 2015 during the Mediterranean International Trawl Survey (MEDITS), an international bottom-trawl survey designed to assess abundance and distribution of demersal resources in the Mediterranean Sea [25,26]. The collection of samples for the present study was developed in different locations over trawlable areas (Figure 1) along the coast with a mean depth range of 43–349 m, and anthropogenic influence including aquaculture installations [27]. The trawls were carried out with a bottom-trawl gear (GOC-73) with a cod-end mesh size of 20 mm. Haul duration was of 30 or 60 min for those stations located at <200 m or >200 m depth, respectively [25,26]. Afterwards, the capture was sorted on board and specimens of two cephalopod mollusks, *Alloteuthis media* and *Abralia veranyi*, and one decapod crustacean, *Plesionika heterocarpus*, were frozen at 20 °C until processing. One eye was dissected from each frozen specimen, preserved in RNA isolation reagent TriReagent^®®^ (Sigma-Aldrich, Merck KGaA, Darmstadt, Germany) and stored at −80 °C, pooling up to six individuals.

### 2.2. Isolation and Sequencing of Betanodavirus RNA2 Genomic Fragments

Total RNA was isolated from frozen ocular tissue using the Quick-RNA™ MiniPrep Plus Kit (Zymo Research, Irvine, CA, USA) following the manufacturer’s instructions. The first strand of cDNA was synthesized by reverse transcription using the BioScript reverse transcriptase (Bioline Meridian Life Science, Memphis, TN) and random primers (Invitrogen) according to the manufacturer’s instructions. Subsequent Nested-PCR amplification of a fragment of the *T4* region of RNA2 of strains RGNNV or SJNNV was performed using the specific primers shown in Table 1. Negative and positive controls were always included in the reactions. The positive control was a cDNA from an experimentally infected fish with RGNNV [11]. Sanger sequencing of the amplified fragments was performed at the “Servicio de Apoyo a la Investigación” of the University of Murcia using an ABI Prism 3130 sequencer (Applied Biosystems, Walthan, MA, USA). Chromas 2.6 and MEGA7 [28] were used to inspect sequences for quality trimming and consensus assembling.

### 2.3. Phylogenetic Analysis

Reference sequences used to assign NNV strain and the corresponding GenBank accession numbers were SJNNV (D30814), BFNNV (D38635), RGNNV (D38636 and EF558369), TPNNV (D38637). We also included crustacean NNV reference sequences *Penaeus vannamei* (Pv)NNV (NC_014977) and *Macrobrachium rosenbergii* (Mr)NNV (NC_005095). The GenBank database was explored in search of sequences from studies describing detection of the RNA2 genomic fragment of NNV in shellfish of the Mediterranean Sea or fish from adjacent areas. Retrieved sequences were aligned and inspected manually in MEGAX [31], which was further used for sequence alignment, substitution model selection and phylogenetic tree construction. Aligned sequences were trimmed and duplicate redundant sequences obtained from the same species and ecosystem eliminated. The Kimura-2 parameter substitution model [32] was selected according to the lowest AIC (Akaike Information Criterion). Bootstrap consensus phylogenetic trees were inferred using the Maximum Likelihood method from 1000 replicates including all positions [33]. The initial tree for the heuristic search was obtained automatically by applying Neighbor-Join and BioNJ algorithms to a matrix of pairwise distances, estimated using the Maximum Composite Likelihood (MCL) approach, and then selecting the topology with superior log likelihood value.

## 3. Results and Discussion

### 3.1. Detection of RGNNV in Squid and Shrimp Species Collected in the Northern Alboran Sea (ALB)

**Figure 2:**
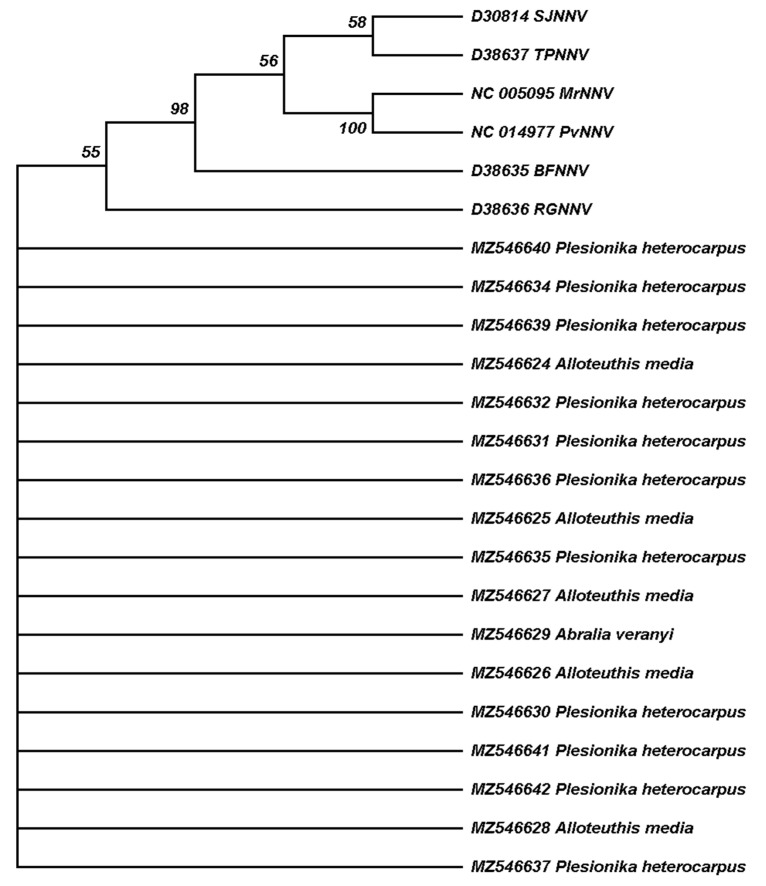
Maximum Likelihood phylogenetic tree reconstruction based on a 102 bp genomic The GenBank accession number is indicated for each sequence. Black symbols correspond to sequences obtained from shellfish and white symbols to sequences obtained from fish, whereas circles indicate reference sequences and triangles sequences obtained in this study. Branches corresponding to 50% or higher bootstrap replicates are shown, indicating the percentage of replicate trees in which the associated sequences clustered together in the bootstrap test.

The Mediterranean Sea is connected to the Atlantic Ocean through the Strait of Gibraltar, and thus the most western Mediterranean basin, the ALB, is a transition area between Atlantic and Mediterranean waters. The ALB is highly biodiverse and has been subject to exploitation through intensive fishing for centuries. Concerning demersal natural resources, the main target species of bottom trawlers in ALB are European hake, mullets, octopus and shrimps [34]. Industrial aquaculture activity in ALB has been ongoing for the last four decades, and in 2018, the main cultured species in the northern ALB were sea bass and mussel [27]. To assess the presence of NNV reservoirs in the ALB, marine invertebrates were collected in spring 2015 during the MEDITS survey. Analyzed species were two cephalopod mollusks, *Alloteuthis media* (Linnaeus, 1758) and *Abralia veranyi* (Rüpell, 1844), and one species of decapod crustacean, *Plesionika heterocarpus* (A. Costa, 1871). RT-PCR and sequencing using RGNNV and SJNNV strains-specific primers (Table 1) detected NNV RNA2 in the RNA extracted from ocular tissue of the three species analyzed (Table 2) and in eight of the nine locations studied (Figure 1). Considering NNV is known to infect crustaceans, our analytical effort was focused on *P. heterocarpus* (Table 2), screening a total of 143 shrimp specimens and 15 squid. NNV was detected in five out of six trawls in which *P. heterocarpus* was collected, representing 35% of the pooled individuals that were analyzed. For the screening of NNV reservoirs in Cephalopoda, *A. media* specimens were collected in two trawls and *A. veranyi* in one trawl. We detected NNV in 73% of the squid specimens that were analyzed. We obtained a total of 17 sequences from RNA extracted from the pooled ocular tissue, 11 isolated from shrimp eyes and 6 from squid eyes. Sequence similarity to reference sequences of the NNV genera *Betanodavirus* and to unclassified prawn nodaviruses indicates that all genomic fragments isolated from invertebrate animals in the ALB belong to the RGNNV strain, as illustrated by sequence clustering (Figure 2).

In the Mediterranean basin, two different strains of betanodaviruses have been reported, the RGNNV and the SJNNV strains, together with their reassortants [2]. According to this study, in the ALB only the RGNNV strain is detected in the three invertebrate species analyzed. To date, mainly the strains RGNNV and BFNNV have been detected in invertebrate hosts [2]. The BFNNV strain has only been detected in invertebrates in Asian waters, whereas the RGNNV is the most frequently detected strain in Mediterranean invertebrates [2]. Our analyses were performed to detect the RNA2 *T4* fragment of RGNNV and SJNNV, which are the most abundant betanodaviruses, using a set of primers designed to detect both strains [29,30]. However, in our study, we only found RGNNV. To date, the SJNNV/RGNNV reassortant has been mainly identified in fish species from Italy or Greece [35,36]. Interestingly, the reassortant SJNNV/RGNNV has been recently detected in mussels collected in the Adriatic Sea [9]. The RGNNV/SJNNV reassortants exhibit a slightly modified SJNNV capsid protein [4], thus, we can assure that this reassortant was not detected in the present study. We cannot discard the presence of the SJNNV/RGNNV reassortant in ALB invertebrates, as we only analyzed RNA2. Although the ability of the virus to replicate in invertebrate cells is not clear enough, certain studies suggest that the virus is bioaccumulated in bivalve tissues and can shed through water and fecal matter [13]. Whether this issue is due to the ability of RGNNV to infect invertebrate cells without causing disease, as has also been described for RGNNV in certain fish species [37], or due to the virus avoiding inactivation in the invertebrate tissues and being bioaccumulated, is something that demands further study.

### 3.2. Distribution of RGNNV Reservoirs in Invertebrate Species of the Mediterranean Sea

After detecting the RGNNV strain in invertebrate species of the ALB, we compiled our data in Table 3 with other studies reporting NNV in shellfish species collected in the Mediterranean Sea [9,20,22,23,24]. A total of eight invertebrate marine species have been documented to host RGNNV, and they are categorized in a broad range of taxonomic classes (Bivalvia, Gastropoda, Cephalopoda and Malacostraca). All invertebrate animals reported as hosts of RGNNV are widely distributed in the Mediterranean Sea. In the case of bivalve mollusks, the RGNNV strain has been detected in mussels and clams that were collected in Sicily, in the Adriatic Sea and in the Aegean Sea [9,20,22]. For gastropod mollusks, the virus was detected in a single species, *Stramonita haemastoma*, collected in Greece [22]. Concerning cephalopod mollusks, in this study we report for the first time in the Mediterranean basin detection of RGNNV in the two species of squid that had been collected in the ALB. A study reported betanodaviruses in four specimens of wild and cultured *Octopus vulgaris* [23] without identifying the NNV strain. In relation to arthropods, we also report that RGNNV was detected in the ALB in the shrimp *P. heterocarpus*, with a previous study detecting the virus in blue crab collected in the Levantine Sea [24]. In summary, genomic RNA2 of RGNNV has been detected in the nervous system (nerve or ocular tissue) or the digestive system (hepatopancreas) of marine shellfish species collected in the western Mediterranean Sea (ALB), in the central Mediterranean Sea (Sicily, Aegean and Adriatic Seas), and in the eastern Mediterranean Sea (Levantine Sea) (Table 3).

**Figure 3:**
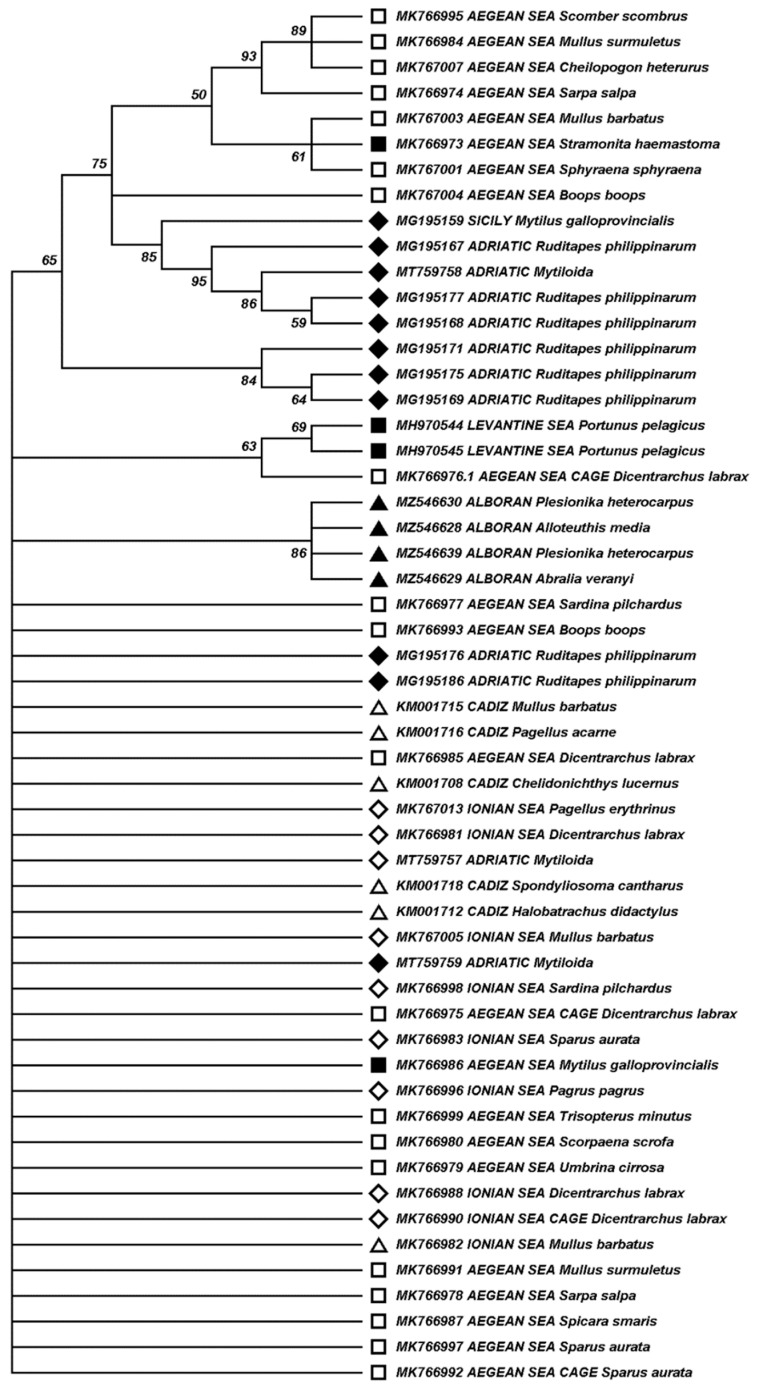
Clustering of RGNNV sequences obtained from shellfish and fish collected in the Mediterranean Sea and adjacent Atlantic waters. GenBank accession numbers and RGNNV host species are indicated for each sequence. Black symbols correspond to sequences obtained from shellfish and white symbols to sequences obtained from fish. Sequences obtained from samples collected in western Mediterranean (ALB) or Atlantic adjacent waters (Gulf of Cadiz) are indicated with triangles; from central Mediterranean (Sicily, Ionian and Adriatic Sea) with diamonds; and from the eastern Mediterranean (Aegean and Levantine Sea) with squares. Branches corresponding to 50% or higher bootstrap replicates are shown, indicating the percentage of replicate trees in which the associated sequences clustered together in the bootstrap test. There were a total of 143 positions in the final dataset.

The mechanism behind the presence of RGNNV in Mediterranean invertebrate species is unknown. Molecular interactions may be important if there is specificity in the virus host entry route to cells of invertebrate hosts. Substitution of certain amino acids of the capsid protein, Ser247Ala and Ser270Asp, has been reported to reduce virulence for different viral strains supporting the role of these positions in NNV virulence for fish [38,39]. In invertebrates, such as squid or shrimp species analyzed in this study, infection may follow mechanisms such as clathrin-mediated endocytosis, a route for uptake of virus-like particles mimicking NNV [40]. Specific molecular virus–host interactions may be less important in shellfish, as viral particles may be accumulated though filtering water contaminated by fish of the same habitat. The infection and/or bioaccumulation of RGNNV in invertebrates makes the infection of fish species that eat invertebrates in the wild feasible, which in turn can be vehicle of the virus into cultured marine fish. In an attempt to decipher if the distribution of RGNNV in Mediterranean invertebrates is driven by molecular, specific, virus–host interactions or by ecosystemic factors, we aligned all nucleotide sequences of the RGNNV RNA2 fragment reported for invertebrate species collected in the Mediterranean Sea. Certain sequences obtained from wild or farmed fish species collected in the Mediterranean Sea and collected together with shellfish species from which RGNNV had been detected [22] were also included in our analysis, as well as sequences obtained from wild fish from the Gulf of Cadiz [29], as this area is highly connected to the Mediterranean Sea through the Strait of Gibraltar. Table 4 details the features of the fish species from which the sequences were obtained, including the biological niche, illustrating that trophic behavior poses a risk of transmission through feeding. A dendrogram was constructed through phylogenetic analysis (Figure 3), including sequences obtained in the present study or compiled in Table 3 and Table 4, and eliminating duplicate redundant sequences obtained from the same species and ecosystem. No clustering of nucleotide sequences detected in related taxa was found, indicating an apparent lack of specificity in the viral–host interaction through the compared fragment encoding the *T4* region of RGNNV capsid protein. In addition, there was no apparent strong clustering in relation to the collection area within the Mediterranean Sea of the invertebrate species hosting RGNNV. Our data suggest that the RGNNV virus is currently distributed throughout the Mediterranean basin, and support the original hypothesis that the RGNNV is being widely transmitted between invertebrates and fish species and between different areas and ecosystems.

Overall, our data demonstrated the presence of RGNNV in squid and shrimp species collected in the Alboran Sea and showed that the RGNNV is being widely transmitted in the Mediterranean Sea between invertebrates and fish species and between different areas and ecosystems.

## Figures and Tables

**Table 1:** Primer sequences used for detection of a fragment of RNA2 encoding the capsid protein of NNV strains SJNNV and RGNNV.

PCR	Primer	Oligonucleotide Sequence (5′-3′) ^a^	Reference
First PCR	Noda F2.3	CRTCYCTYGAGACACCTGA	[29]
	Noda-Rev2	CSCCAWCTGTGAAYGTMTTGT	[30]
Nested PCR	Noda-Fw1	CCTGARGASACCACCGCTCCMAT	[30]
	Noda R3.2	TGTARTCAATGGRCARCGG	[29]

^a^ Degenerations: R = G, A; Y = T, C; S = G, C; W = A,T; M = A,C.

**Table 2:** Detection of NNV in shrimp and squid collected from the Alboran Sea (ALB) for this study.

Class ^a^	Species	Collection Date	Collection Area ^b^	Mean Depth (m)	Total Individuals ^c^	Detection of NNV (%) ^d^
Malacostraca (n 143, 6 trawls)	*Plesionika heterocarpus*	23 April 2015	Málaga (L01)	119	16	0
		24 April 2015	Estepona (L06)	139	36	22
		26 April 2015	Marbella (L14)	236	46	24
		02 May 2015	Castell de Ferro (L40)	166	20	80
		04 May 2015	Almería (L49)	327	13	31
		06 May 2015	Cabo de Gata (L57)	251	12	100
Cephalopoda (n 15, 3 trawls)	*Alloteuthis media*	23 April 2015	Torremolinos (L02)	85	5	80
		26 April 2015	Fuengirola (L16)	43	4	100
	*Abralia veranyi*	27 April 2015	Vélez-Málaga (L22)	349	6	50

^a^ Total number of individuals analyzed and trawls are indicated in brackets. ^b^ The trawl number is indicated in brackets after letter L. ^c^ Number of individuals analyzed per trawl. ^d^ Percentage of detection of NNV is indicated in relation to the total number of individuals screaned.

**Table 3:** Detection of NNV in shellfish collected in the Mediterranean Sea.

Classification ^a^	Species	Collection Area	Tissue ^b^	RNA2 Sequence ^c^	Strain	Reference
Mollusca Bivalvia						
Mytiloida Mytilidae	*Mytilus galloprovincialis* *Xenostrobus securis*	Greece, Aegean Sea.	n.i.	MK766986.1	RGNNV	[22]
		Sicily, Italy.	Hepatopancreas	MG195159.1	RGNNV	[20]
		Italy, Adriatic Sea.	Hepatopancreas	MT759755-MT759756	SJNNV	[9]
		Italy, Adriatic Sea.	Hepatopancreas	MT759757-MT759760	RGNNV	[9]
Veneroida, Veneridae	*Ruditapes philipinarum*	Italy, Adriatic Sea.	Hepatopancreas	MG195167.1- MG195186.1	RGNNV	[20]
Mollusca Gastropoda						
Neogastropoda Muricidae	*Stramonita haemastoma*	Greece, Aegean Sea.	n.i.	MK766973.1	RGNNV	[22]
Mollusca Cephalopoda						
Octopoda Octopodidae	*Octopus vulgaris*	Italy, Ligurian Sea	Skin lession	n.i.	n.i.	[23]
Myopsida Loliginidae	*Alloteuthis media*	Spain, Alboran Sea.	Ocular tissue	MZ546624-MZ546628 ^d^	RGNNV	This study
Oegopsida Enoploteuthidae	*Abralia veranyi*	Spain, Alboran Sea.	Ocular tissue	MZ546629	RGNNV	This study
Arthropoda Malacostraca						
Decapoda Pandalidae	*Plesionika heterocarpus*	Spain, Alboran Sea.	Ocular tissue	MZ546630-MZ546642	RGNNV	This study
Decapoda Portunidae	*Portunus pelagicus*	Israel, Levantine Sea.	Nerve tissue	MH663494.1, MH663495.1, MH970545.1, MH970544.1	RGNNV	[24]

^a^ Phylum, Class, Order, Family. ^b^ n.i. not indicated. ^c^ GenBank accession number. ^d^ Excluding MZ546633 and MZ546638.

**Table 4:** Mediterranean fish species used to assess distribution of RGNNV reservoirs in shellfish through phylogenetics.

Classification ^a^	Species	RNA2 sequence ^b^	Collection Area ^c^	Reference ^d^	Biological Trophic Niche	Reference
Scorpaeniformes						
Triglidae	*Chelidonichthys lucernus*	KM001708	Gulf of Cadiz	[29]	Fish, crustacean and mollusks	[41]
Scorpaenidae	*Scorpaena scrofa*	MK766980	Aegean Sea	[22]	Fish, crustacean and mollusks	[42]
Batrachoidiformes						
Batrachoididae	*Halobatrachus didactylus*	KM001712	Gulf of Cadiz	[29]	Fish and crustacean	[43,44]
Beloniformes,						
Exocoetidae	*Cheilopogon heterurus*	MK767007	Aegean Sea	[22]	Not determined	
Clupeiformes						
Clupeidae	*Sardina pilchardus*	MK766977	Aegean Sea	[22]	Fish eggs, crustacean eggs, copepods, decapods, cirripedes, dinoflagellates and diatoms.	[45,46]
		MK766998	Ionian Sea	[22]		
Gadiformes						
Gadidae	*Trisopterus minutus*	MK766999	Aegean Sea		Fish and crustacean	[47,48]
Perciformes						
Sparidae	*Boops boops*	KM767004	Aegean Sea	[22]	Mainly on crustaceans, also planktophagous	[49]
		MK766993	Aegean Sea	[22]		
	*Sparus aurata*	MK766992	Aegean Sea (Farm)	[22]	Shellfish, including mussels and oysters	[50]
		MK766983	Ionian Sea	[22]		
		MK766997	Aegean Sea	[22]		
	*Pagellus erithrinus*	MK767013	Ionian Sea	[22]	Mainly benthic invertebrates and small fishes, also omnivorous	[50]
	*Spondyliosoma cantharus*	KM001718	Gulf of Cadiz	[29]	Seaweeds and small invertebrates (crustaceans)	[50]
	*Pagellus acarne*	KM001716	Gulf of Cadiz	[29]	Worms, mollusks and small crustaceans, also omnivorous	[50,51]
	*Pagrus pagrus*	MK766996	Ionian Sea	[22]	Crustacean, fishes and mollusks	[50,52]
	*Spicara smaris*	MK766987	Aegean Sea	[22]	Zooplanktivorous, mainly fish larvae	[53]
	*Sarpa salpa*	MK766974	Aegean Sea	[22]	Plankton feeders and herbivorous	[54]
		MK766978	Aegean Sea	[22]		
Moronidae	*Dicentrarchus labrax*	MK766976	Aegean Sea (Farm)	[22]	Fish, crustaceans and mollusks	[55]
		MK766975	Aegean Sea (Farm)	[22]		
		MK766990	Ionian Sea (Farm)	[22]		
		MK766985	Aegean Sea	[22]		
		MK766981	Ionian Sea	[22]		
		MK766988	Ionian Sea	[22]		
Scianidae	*Umbrina cirrosa*	MK766979	Aegean Sea	[22]	Fish and benthic invertebrates, also omnivorous	[56]
Mullidae	*Mullus barbatus*	KM001715	Gulf of Cadiz	[29]	Small benthic crustaceans, worms and mollusks	[57]
		MK767003	Aegean Sea	[22]		
		MK767005	Ionian Sea	[22]		
		MK766982	Ionian Sea	[22]		
	*Mullus surmuletus*	MK766984	Aegean Sea	[22]	Fish, crustacean and mollusks	[58]
		MK766991	Aegean Sea	[22]		
Scombriformes						
Sphyraenidae	*Sphyraena sphyraena*	MK767001	Aegean Sea	[22]	Fish, cephalopods and crustaceans	[59]
Scombridae	*Scomber scombrus*	MK766995	Aegean Sea	[22]	Zooplankton and small fishes	[60]

^a^ Phylum Order, Family. ^b^ GenBank accession number. ^c^ Area of collection of specimens from which RGNNV RNA2 sequence were obtained. ^d^ Reference detecting RNA2 of RGNNV in shellfish and indicated fish species.

## Data Availability

The GenBank accession numbers for partial sequences of NNV RNA2 obtained in this study are MZ546624-MZ546642, excluding MZ546633 and MZ546638.
